# Editorial: Transcriptional Regulation of Glucose Metabolism: Gaps and Controversies

**DOI:** 10.3389/fendo.2019.00629

**Published:** 2019-09-18

**Authors:** Antonio Brunetti, Biagio Arcidiacono, Daniela Patrizia Foti, Robert K. Semple

**Affiliations:** ^1^Department of Health Sciences, University “Magna Graecia” of Catanzaro, Catanzaro, Italy; ^2^Centre for Cardiovascular Science, University of Edinburgh, Edinburgh, United Kingdom

**Keywords:** glucose homeostasis, gene transcription, insulin resistance, insulin signaling, microRNAs

In mammals, glucose homeostasis is strictly regulated to guarantee energy supply to vital organs and tissues. However, abnormalities in glucose homeostasis may occur in several common endocrine/metabolic disorders, such as obesity, diabetes, and the metabolic syndrome (1–3). In recent decades, much work has been devoted to elucidating the molecular and cellular mechanisms underpinning glucose homeostasis, and both ubiquitous and tissue-specific transcription factors, such as PPARγ, ChREBP, FOXO1, and PDX-1 (among many others), have been identified to be key (4–9). In addition, new areas of investigation have recently been developed, following the observation that microRNAs (miRNAs) play a role in the modulation of glucose metabolism (10, 11). This Research Topic aimed to add pieces to the mosaic of knowledge about the molecular mechanisms implicated in the regulation of glucose metabolism.

Chiefari et al., following previous studies from the same research group (12, 13), describe the central role of the high-mobility group A1 protein, HMGA1, in the transcriptional regulation of genes and gene networks implicated in insulin receptor signaling and glucose homeostasis. In their review, the Authors provide an overview of HMGA1's metabolic roles, summarizing data that support an important role for this architectural chromatin factor in the regulation of genes implicated in the maintenance of glucose metabolism and metabolic control (14). From a clinical standpoint, the Authors emphasize the role of HMGA1 in glucose metabolism by highlighting the association of the *HMGA1* gene locus with susceptibility to a variety of clinical conditions, including rare insulin resistance syndromes (15), type 2 diabetes (16), and the metabolic syndrome (17). The authors make the case that HMGA1 may constitute a link between glucose metabolism and other biological processes in which HMGA1 is involved, such as cell proliferation and differentiation, that need to be sustained by cell energy.

Oriente et al. summarize the importance of another transcription factor which plays a role in the regulation of energy homeostasis and metabolism, namely Pbx-regulating protein-1 (Prep1). This acts in the network that controls pancreas development and differentiation. It is interesting to note that mice with a partial functional loss of Prep1 have smaller pancreatic islets and reduced insulin secretion on one hand, while, on the other, they show higher insulin sensitivity in peripheral tissues, and exhibit protection from both streptozotocin-induced diabetes and diet-induced steatohepatitis. The suggestion is advanced that Prep1 might be a new target for improving and treating metabolic diseases.

Several studies have described the association of uric acid (UA) and insulin resistance (18, 19). Using HUVEC cells as an *in vitro* model of the endothelium, Tassone et al. investigated the effects of UA on insulin signaling. The Authors show that, in endothelial cells, UA promotes binding of ENPP1, an insulin receptor (IR) inhibitor, to the IR, leading to impairment of insulin signaling, and inhibition of insulin-induced Akt/eNOS activation. The data support the hypothesis that, by interfering with IR signaling, UA can cause insulin resistance, and this may contribute to the link between hyperuricemia, diabetes, and vascular damage.

In recent years, huge attention has been paid to the potential of so called “nutraceuticals” to counteract high-fat diet (HFD)-dependent metabolic disorders. Many studies have reported the beneficial impact of a Mediterranean diet on glucose homeostasis (20, 21), and Lombardo et al. now advance this by showing that a specific nutraceutical compound derived from extra-virgin olive oil, oleacein, can improve lipid profile, insulin resistance, and glucose homeostasis in HFD mice. Remarkably, olive oil polyphenols are among the compounds with EU approval for claiming health benefit (EC regulation 432/2012). As the Authors point out, further studies are now required to assess the potential of oleacein in preventing and treating glucose intolerance-related diseases in humans.

Research on miRNAs constitutes a hot topic for research in biomedicine, and abnormal expression of miRNAs is associated with a variety of human diseases (22). However, while the identification of miRNAs in the various pathological contexts is rising exponentially, the discovery of validated miRNA-target genes lays far behind, representing a big challenge for future investigations on the pathophysiology and therapy of diseases.

Sebastiani et al. study the expression profiles of circulating miRNAs in gestational diabetes mellitus (GDM). The Authors report that one miRNA, miR330-3p, is upregulated in pregnant women with GDM compared to healthy non-diabetic pregnant women. GDM patients with higher levels of circulating miR-330-3p moreover showed lower levels of insulinemia and a more adverse course of GDM compared to those with lower miR-330-3p. Through computational analysis, miR330-3p was demonstrated to be a modulator of genes implicated in β-cell proliferation and insulin secretion. Therefore, the Authors suggest that miR-330-3p may be a biomarker of GDM outcome as well as a therapeutic target.

The contribution from Capuani et al. addresses a different aspect of pancreatic β-cell homeostasis, focusing on the incretin hormone glucagon-like peptide 1 (GLP-1), and its action on β-cell insulin secretion through the modulation of expression of specific miRNAs which increase glucose and GLP-1-stimulated insulin release. The authors suggest that some miRNAs (namely, miR-132 and miR-212) may be implicated in the pathogenesis of diabetes mellitus, and also that modulation of miRNA expression by GLP-1 in the liver appears to reduce hepatic lipid accumulation and liver steatosis. Based on these findings, the Authors emphasize how a deeper understanding of the GLP-1/miRNA pathway could contribute to development of novel therapeutic strategies for prevention and treatment of diabetes and hepatosteatosis.

Mirra et al. review the most recent literature in order to highlight miRNAs that are able to switch off expression of genes implicated in the TCA cycle and to activate genes involved in aerobic glycolysis. The authors also describe the association between miRNAs and metabolism, by evaluating the effects of these small mRNA molecules on insulin signaling, on glucose uptake and on the mitochondrial oxidative metabolism. Future investigation aimed at elucidating the clinical relevance of miRNAs, in this context, is highlighted also in this review. On the same subject, Mobbs elegantly illustrates how transient or prolonged exposure to glucose may give rise to changes in the expression of genes that promote glycolysis or inhibit genes of alternative metabolic pathways, such as the pentose and fatty acid oxidation pathways, and the TCA cycle, also known as the Krebs cycle. This phenomenon, known as transcriptional “hysteresis,” may explain several pathological consequences of high glucose exposure of pancreatic β-cells, such as an increase in insulin secretion, diabetes, and the persistence of diabetic complications.

In conclusion, the present Research Topic attests to the diversity of current research efforts to unpick the regulatory mechanisms at play in glucose metabolism. It illustrates some emerging themes and introduces new players including proteins and miRNAs that could not only add insights into the regulation of genes, enzymes, and/or intracellular pathways involved in glucose metabolism, but which may also emerge as novel biomarkers and therapeutic targets for improving glycemic control.

## Author Contributions

All authors listed have made a substantial, direct and intellectual contribution to the work, and approved it for publication.

### Conflict of Interest Statement

The authors declare that the research was conducted in the absence of any commercial or financial relationships that could be construed as a potential conflict of interest. The handling editor declared a shared affiliation, though no other collaboration, with one of the authors RS at the time of the review.
